# Reproductive Performance and Milk Composition of Sows Fed Diets Supplemented with an Immunomodulator

**DOI:** 10.3390/ani15101427

**Published:** 2025-05-15

**Authors:** Mark J. Estienne, Jung W. Lee, R. Tyler Niblett, Brooke D. Humphrey, H. James Monegue, Merlin D. Lindemann

**Affiliations:** 1 Virginia Tech-Tidewater Agricultural Research and Extension Center, Suffolk, VA 23437, USA; mestienn@vt.edu (M.J.E.);; 2 Department of Animal and Food Sciences, University of Kentucky, Lexington, KY 40546, USA; 3 Phibro Animal Health Co., Teaneck, NJ 07666, USA

**Keywords:** immunomodulator, sow, lactation, feed intake, litter size

## Abstract

A cooperative study involving 189 litters from 114 sows (initial BW of 200.8 ± 37.1 kg) at two experiment stations was conducted to investigate the effects of dietary supplementation with OmniGen-AF (OG) (Phibro Animal Health Co., Teaneck, NJ, USA), a nutritional product formulated to improve immune function of animals, on sow reproductive performance and milk composition. Dietary treatments were (1) corn–soybean meal-based control diets or (2) control diets supplemented with OG at 0.75% (~9 g of OG/100 kg BW/d). Supplementation of diets with OG resulted in lesser (*p* < 0.05) BW changes of sows during lactation (−12.1 vs. −8.2 kg). Litter sizes for control and OG-fed sows were similar, but sows fed OG-based diets had greater (*p* < 0.05) litter weight for total born (18.3 vs. 19.3 kg) and weaned (63.2 vs. 67.0 kg) and lactation litter gain (47.8 vs. 50.7 kg). Lactation feed intake for the controls and OG-fed sows (5.32 vs. 5.52 kg/d, respectively) did not differ. Supplementing diets with OG increased lactose content (5.78 vs. 5.84%; *p* = 0.05) and reduced protein content (4.77 vs. 4.68%; *p* = 0.04) in sow milk. In conclusion, dietary supplementation with OG at 0.75% reduced weight loss during lactation and improved litter weights with marginal effects on the milk composition of sows.

## Simple Summary

The aim of this study was to evaluate a commercially available product in reproducing pigs that has been demonstrated in dairy cows to increase milk yield and milk composition. The results herein demonstrated no effect on sow prolificacy but did demonstrate changes in milk composition and greater litter weight born, litter weight weaned, and litter weight gain.

## 1. Introduction

OmniGen-AF^®^ (OG) (Phibro Animal Health Co., Teaneck, NJ, USA) is a nutritional product formulated to improve the immune function of animals; it contains a proprietary mixture of silicon dioxide, calcium aluminosilicate, sodium aluminosilicate, brewers dehydrated yeast, mineral oil, calcium carbonate, rice hulls, a niacin supplement, biotin, d-calcium pantothenate, choline chloride, vitamin B-12 supplement, thiamine mononitrate, pyridoxine hydrochloride, riboflavin 5-phosphate, and folic acid. Previous studies have demonstrated that OG reduced the negative effects of stressors on dairy cow performance [1,2]. Moreover, when fed to cattle, the supplement reduced the inflammation response and enhanced immune health [3,4]. Furthermore, supplementation of dairy cows during the transition period [5,6] has resulted in increased milk yield after calving, with reports [7,8] that milk fat is increased. Although it is widely used in the dairy industry, there is a lack of information pertaining to the supplementation of OG in sow diets. Therefore, the objective of this study was to evaluate the effects of supplemental dietary OG on both sow reproductive performance and on milk composition during early and late lactation.

## 2. Materials and Methods

### 2.1. Ethics Statement

The Institutional Animal Care and Use Committees at the University of Kentucky (Lexington, KY, USA) (IACUC 2019-3227) and Virginia Tech (Blacksburg, VA, USA) (18-193 AREC) reviewed and approved all animal procedures employed in this experiment.

### 2.2. Animals, Dietary Treatments, and Housing Conditions

A total of 114 gestating sows (80 [Yorkshire × Landrace] × Large White sows from the University of Kentucky and 34 [Yorkshire × Landrace or Berkshire] × Duroc sows from Virginia Tech) (initial BW of 200.8 ± 37.1 kg) were allotted to one of two treatments in a completely randomized design that was balanced for breed, actual biological parity, and bodyweight (BW).

Corn–soybean meal-based basal diets were formulated to meet or exceed NRC [9] requirement estimates during gestation and lactation. A common vitamin and trace mineral premix was used at the two stations. Dietary treatments were mixed and prepared individually and fed in mash form and were (1) a basal diet (control) supplemented with 0% OG and (2) the basal diet supplemented with 0.75% OG (Table 1). The inclusion rate was calculated to supply sows with about 9 g of OG/100 kg BW/d; this mean rate of 0.75% was divided into inclusion rates of 1.0% and 0.5% in early and late lactation, respectively, based on higher feed intake in late lactation. Sows were fed OG-based diets at least 21 d prior to breeding and 1.9 kg/d of their assigned diet during gestation, and after farrowing, they were provided feed in a step-up basis until the sows were on ad libitum access by 5 to 10 d post farrow. The sows were fed the appropriate diets during lactation prior to being weaned and bred for the current study; this ensured that all females fed OG would receive the product prior to ovulation for the litters counted in the study. The respective diets were fed continuously until the sows ended the study.

Sows were checked for estrus daily in the presence of a mature boar and bred using artificial insemination 0 and 24 h after first detection of the lordosis response. During gestation, pregnant sows were housed individually in gestation crates (0.57 × 2.13 m) in environmentally controlled rooms. Ventilation was automatically controlled to keep proper air exchange, and temperature was maintained at a minimum of 20 °C by a thermostatically regulated heater in the room.

Pregnant sows were moved to farrowing crates (1.52 × 2.13 m) in an environmentally controlled room 3 to 5 d before farrowing. The crates had a plastic-coated woven wire floor area with heat lamps for piglets and were equipped with a drinking nipple for sow and pigs and a feed trough for sows.

### 2.3. Measurements

The BW of sows was measured at breeding, d 110 of gestation, within 24 h post farrowing, and at weaning. Within 24 h postpartum, total born, born alive, stillborn, and dead pigs were recorded. Within 3 d after birth, piglets were cross-fostered within treatment to balance the number of suckling piglets at UK. At VT, litter integrity was maintained as much as possible, but cross-fostering within treatment was carried out if necessary. Individual pig weights were measured at birth and weaning, and daily feed intake of lactating sows was recorded. Because of different weaning ages across litters (X = 20.7 ± 2.2 d), the piglet BW at weaning was normalized by the following equation:

Adjusted weaning weight = BW after cross-fostering + [(BW at weaning − BW after cross-fostering)/weaning age] × 21.

At UK, milk samples were collected from teats 3 and 4 on the sow’s right side at d 5 to 7 (early lactation) and d 14 to 17 (late lactation) after farrowing. Milk composition analysis was conducted using a Foss Milkoscan™ FT+ device (FOSS Electric, Eden Prairie, MN, USA). The gross energy content of the complete milk was calculated from the concentration of lactose, fat, and protein, which contributed 16.4, 38.9, and 23.8 KJ/g, respectively [10].

### 2.4. Statistical Analysis

All data were subjected to ANOVA for a completely randomized design using the GLM procedure in SAS, version 9.4 (SAS Institute Inc., Cary, NC, USA). Individual sow and litter served as the experimental unit. The statistical model included the effects of OG, station, parity on test, and OG × parity on test. For milk composition analysis, the model included the effects of treatment, lactation stage (early or late), and treatment × lactation stage. Data were examined for outliers outside approximately the 10th and 90th percentiles of the data for the responses in order to reduce the impact of abnormal values on the ability to detect potential treatment differences. The majority of the values examined as potential outliers were ultimately retained in the dataset because when the entirety of the observations for those experimental units were assessed, they were deemed to be within that which is observed in a normal population. However, when an observation was deemed to be an outlier, all data for that sow were removed. There were no outliers removed that were identified initially based on liveborn litter size, number of stillborns, and percentage mortality from birth to weaning. Outliers were initially identified and removed based on sow BW changes, including abnormally low gestation gain (n = 1 [one 0% OG sow]) and high lactation gain (n = 5 [two 0% OG sows and three 0.75% OG sow]). For litter sizes, outliers were initially identified and ultimately removed based on total born (values not between 7 and 18; n = 5 [four 0% OG sows and one 0.75% OG sow]), mortality during lactation (values > 4; n = 1 [one 0% OG sow]), and weaning-to-estrus interval (values > 20 d; n = 4 [three 0% OG sow and one 0.75% OG sow]). The results of the analyses are reported as least square means, and statistical differences were considered significant at *p* < 0.05 with tendencies at 0.05 < *p* < 0.10.

## 3. Results

During a preliminary analysis, a model that included OG, station, and OG × station did not detect any effects of the interaction term on any response variable measured. Thus, the OG × station interaction term was removed from the model, and parity on test as well as OG × parity on test effects were added to the final model. Parity on test represents the number of parities or litters for which the treatments were received and not the actual biological parity, as some sows had more than one litter during the study. Table 2 summarizes the effect of OG, station, and parity on test for sow and litter performance measures. Not surprisingly, there were effects (*p* < 0.05) of station on sow BW at breeding, pre- and postfarrow and weaning BW, BW changes during gestation and lactation, lactation feed intake, and the weaning-to-estrus interval. Moreover, there were tendencies for effects of station on liveborn pigs (*p* = 0.085), number weaned (*p* = 0.064), and litter BW gain (*p* = 0.097). There were also anticipated effects (*p* < 0.05) of parity on test on BW at breeding, pre- and postfarrow, and weaning; and the weaning-to-estrus interval as well as tendencies for effects on sow BW change during gestation (*p* = 0.061) and litter BW gain (*p* = 0.060). However, there were no effects or tendencies for effects of OG × parity on test on any response parameters measured except for stillborn pigs (*p* = 0.099).

Supplementation of diets with OG at 0.75% resulted in lesser (*p* = 0.007) weight loss of sows during lactation and an increase (*p* < 0.057) in litter performance, including litter total born weight, litter weaning weight, litter weight gain, adjusted litter weaning weight, and adjusted litter weight gain.

Table 3 summarizes the effects of OG supplementation of diets, stage of lactation (early or late), and OG × stage of lactation on milk composition of sows. There were no effects of OG × stage of lactation on any response measure; thus, the main effects are presented. The percentages of fat, protein, total solids, non-fat solids, and gross energy were greater, and the percentage of lactose was lesser for samples collected early (d 5 to 7) versus late (d 14 to 17) lactation (stage of lactation, *p* < 0.001). Supplementing diets with OG at 0.75% resulted in increased (*p* = 0.057) lactose content and in a reduction (*p* = 0.047) in protein content in the milk of sows compared to the control group.

## 4. Discussion

OmniGen-AF is a proprietary mixture of silicon dioxide, calcium aluminosilicate, sodium aluminosilicate, brewers dehydrated yeast, mineral oil, calcium carbonate, rice hulls, a niacin supplement, d-calcium pantothenate, choline chloride, and a vitamin B-12 supplement. The product is designed to enhance immune function [3,4]. The effects of OG on various response measures demonstrated in this study could likewise involve the immune system. The current study, however, was an applied production-type research endeavor. Defining the exact mechanisms of action responsible for the reported effects will require further experimentation. Moreover, we tested only one level of OG. The inclusion rate of OG fed (9 g/100 kg BW/d), however, approximated those shown to cause physiological responses and effects on growth, at least in cattle [11,12,13]. If repeating the current study, it would be reasonable to explore the dose–response relationship between OG and sow performance.

In the current study, lactating sows fed diets supplemented with OG on an ad libitum basis had numerically but not statistically significant greater daily feed intake than controls. Moreover, compared to controls, OG-fed sows lost 32% less BW during a three-week lactation. Elevated environmental temperatures decrease voluntary feed intake in swine [14], including lactating sows [15]. Perhaps a beneficial effect of OG on feed intake could be more easily demonstrated in sows exposed to elevated environmental temperatures. Consistent with this hypothesis, Hall et al. [12] reported that OG increased feed intake in Holstein cows exposed to heat stress conditions, Gandra et al. [7] reported increased pasture intake of grazing dairy cows in a high-temperature/humidity index environment, and Marques et al. [8] noted an increase in feed intake per unit BW of dairy cows fed a total mixed ration during the hot season.

Despite losing less BW from farrowing to weaning, OG-fed sows had similar weaning-to-estrus intervals and litter sizes compared to sows fed the control diets. This is not surprising because the percentage of BW lost during lactation was relatively small in both groups, being 3.3% in OG sows and 4.8% in control sows. Thaker and Bilkei [16] conducted a study during which 1677 sows on 15 commercial farms were categorized according to lactation BW losses of <5%, 5 to 10%, 11 to 15%, 16 to 20%, and >20%. Weaning-to-estrus intervals were minimized at lactation BW losses of <5% and increased when lactation weight losses increased above 5% for parity 1 sows but not until lactation weight losses exceeded 10% for animals of parity 2 or greater. Lactation weight losses >10% had a negative effect on subsequent farrowing rates and litter sizes.

Sow milk is composed mainly of carbohydrates, lipids, and proteins, with smaller proportions of minerals, vitamins, leukocytes, somatic cells, exosomes, oligosaccharides, and bacteria [17]. In this study, milk samples were collected during early (d 5 to 7 postfarrowing) and late (d 14 to 17 postfarrowing) lactation, and concentrations of fat, protein, and lactose were generally consistent with those previously reported [17]. When comparing early and late lactation composition of milk, significant decreases with time were detected for fat, protein, total solids, non-fat solids, and gross energy. In contrast, lactose concentrations were greater in samples collected during late versus early lactation. The temporal changes in these milk components are completely consistent with those in earlier studies [18,19].

In the current experiment, protein was significantly lesser, and lactose tended to be greater in sows receiving diets with 0.75% OG compared to controls. The differences in these response measures were small, and the biological relevance is unknown. Tan et al. [20] conducted a retrospective study during which sows nursing equal numbers of pigs were classified as high or low lactation performers (n = 15 per group) based on the growth of piglets during a 21 d lactation (4.38 versus 3.24 kg, respectively). On d 18 of lactation in that study, fat (7.59 versus 8.24; *p* = 0.12) and crude protein (5.94 versus 6.14; *p* = 0.23) were lesser and lactose (7.13 versus 6.80; *p* < 0.05) and total solids (28.90 versus 28.16; *p* = 0.10) greater for high- versus low-performing sows. With the exception of total solids, these directional changes in the milk composition of the high-performance sows are consistent with the OG versus control sows in our study. Using the same formula for the calculation of gross energy content as is used in Table 3 herein, the gross energy content of the milk of the high-performance sows was also lesser. It seems counterintuitive that the energy content of the milk for high-performance sows or for the milk of the OG sows that had greater litter weight gain would be lesser; however, changes in the growth of nursing offspring will be a result of either or both changes in milk composition or milk production. Research in dairy cows [5,6,7,8] has demonstrated an increase in milk production and increased milk fat [7,8] and milk protein [8] due to OG supplementation. There would appear to be a species difference in the impact of OG on milk composition when comparing dairy cows and sows, but the increased weight gain of OG-nursed litters would be consistent with the improved milk production observed in dairy cows.

## 5. Conclusions

Supplementation of sow diets with OG at 0.75% resulted in less BW loss during lactation and an increase in litter total born weight, litter weaning weight, litter weight gain, adjusted litter weaning weight, and adjusted litter weight gain, demonstrating a positive effect of feeding OG to gestating and lactating sows on litter performance under the conditions used in this experiment. OmniGen-AF-supplemented sow diets were responsible for an increased content of lactose, whereas they led to reduced protein content in the milk of sows. While the present research was positive, the study does have limitations. Future studies should evaluate dosage levels to determine the optimal dose in swine; using a dosage similar to cattle was successful, but the obvious species differences warrant evaluation of other doses. Additionally, the product was designed as an immunomodulatory product for cattle, and evaluations of various markers of both innate and acquired immunity in swine seems an obvious expansion of response measures to be assessed.

## Figures and Tables

**Table 1:** Formulas and calculated composition of the experiment diets fed during gestation and lactation ^1^ (as-fed basis).

Item	Gestation	Lactation
Ingredient, %		
Corn	77.50	69.57
Soybean meal, 48%	19.00	27.00
L-Lysine-HCl	0.06	0.04
Dicalcium phosphate	1.55	1.60
Limestone	1.00	0.90
Chromax ^6^	0.05	0.05
Choline chloride, 60%	0.10	0.10
Salt	0.50	0.50
Copper sulfate pentahydrate ^2^	0.02	0.02
Trace mineral premix ^3^	0.10	0.10
Vitamin premix ^4^	0.10	0.10
Santoquin	0.02	0.02
Calculated composition		
ME, kcal/kg	3256	3298
Crude protein, %	15.42	18.65
Total Lys, %	0.803	1.004
SID Lys, % ^5^	0.689	0.871
Total Ca, %	0.839	0.840
Total P, %	0.628	0.673

^1^ OmniGen-AF was included in diets at 0 or 0.75%; when included, it replaced corn. ^2^ Supplied 100 ppm Cu to the final diet. ^3^ Supplied per kg of diet: Zn, 110 mg as ZnSO_4_; Fe, 110 mg as FeSO_4_·H_2_O; Mn 50 mg, as MnO; Cu, 18 mg as CuSO_4_·5H_2_O; I, 0.70 mg as CaI_2_O_6_; and Se, 0.30 mg as NaSeO_3_. ^4^ Supplied per kg of diet: vitamin A, 23,402 IU; vitamin D_3_, 5854 IU; vitamin E, 156 IU; vitamin K (as menadione sodium bisulfite complex), 17 mg; riboflavin, 10 mg; pantothenic acid (as d-calcium pantothenate), 52 mg; niacin, 104 mg; vitamin B_12_, 66 μg; d-biotin, 0.57 mg; folic acid, 0.42 mg; and pyridoxine, 10 mg. ^5^ SID = standardized ileal digestible. ^6^ Chromax (Prince AgriProducts, Quincy, IL, USA) supplies chromium picolinate blended in limestone to supply 200 ppb Cr in the complete diet.

**Table 2:** Effects of dietary supplementation with OmniGen-AF (OG) on reproductive performance of sows from the University of Kentucky and Virginia Tech ^1^.

Item	OG, %		SEM ^2^	*p*-Values ^6^		
	0	0.75		OG	Station	Parity on Test
No. of litters	98	91				
Sow body weight, kg						
Breeding	227.2	223.0	5.09	0.686	<0.001	<0.001
Prefarrow ^3^	268.2	261.3	4.30	0.461	<0.001	<0.001
At farrowing	253.8	245.7	4.46	0.184	<0.001	<0.001
At weaning	241.7	237.5	4.45	0.930	<0.001	0.008
Sow weight changes, kg						
Gestation	40.9	38.2	2.50	0.658	0.014	0.061
Lactation	−12.1	−8.2	2.05	0.007	0.003	0.310
Lactation length, d	20.31	20.88	0.31	0.823	0.119	0.064
Lactation feed intake, kg/d	5.32	5.52	0.20	0.188	<0.001	0.230
Weaning-to-estrus interval, d	5.95	5.97	0.24	0.912	<0.001	0.005
Litter size						
Total born	12.97	13.04	0.54	0.402	0.263	0.679
Liveborn	11.69	11.96	0.52	0.488	0.085	0.243
Weaning	10.27	10.52	0.36	0.137	0.064	0.572
Stillborn	1.28	1.06	0.22	0.726	0.210	0.178
Mortality	1.20	1.08	0.24	0.647	0.143	0.492
Survival rate, % ^4^	91.2	91.5	1.60	0.799	0.343	0.396
Litter weight, kg						
Total born	18.3	19.3	0.63	0.042	0.974	0.220
Liveborn	17.1	18.2	0.66	0.135	0.967	0.060
Weaning	61.7	66.9	2.32	0.024	0.124	0.574
Litter weight gain	46.3	50.6	1.93	0.057	0.097	0.577
Adjusted litter weaning weight ^5^	63.2	67.0	2.15	0.020	0.275	0.831
Adjusted litter weight gain ^5^	47.8	50.7	1.72	0.046	0.254	0.978
Piglet weight, kg						
Total born	1.46	1.52	0.04	0.337	0.022	0.666
Liveborn	1.51	1.56	0.04	0.335	<0.001	0.584
Weaning	6.16	6.46	0.18	0.354	<0.001	0.712
Piglet weight gain	4.64	4.89	0.16	0.581	<0.001	0.543
Adjusted piglet weaning weight ^5^	6.29	6.50	0.16	0.291	<0.001	0.848
Adjusted piglet weight gain ^5^	4.76	4.92	0.15	0.505	0.002	0.655

^1^ Data are least squares means of 189 litters; 115 litters from the University of Kentucky (59 and 56 litters for 0 and 0.75% OG, respectively) and 74 litters from Virginia Tech (39 and 35 litters for 0 and 0.75% OG, respectively) for all response measures presented herein except for weaning-to-estrus interval, with least squares means of 165 litters; 105 litters from the University of Kentucky (51 and 54 litters for 0 and 0.75% OG, respectively) and 60 litters from Virginia Tech (31 and 29 litters for 0 and 0.75% OG, respectively) and lactation feed intake, with least squares means of 167 litters; 97 litters from the University of Kentucky (58 and 39 litters for 0 and 0.75% OG, respectively) and 70 litters from Virginia Tech (35 and 35 litters for 0 and 0.75% OG, respectively). ^2^ SEM = standard error of the mean. ^3^ Gestating sows were weighed on d 110 and d 113 at UK and VT, respectively. ^4^ Survival rate was calculated by the following equation: [total number of pigs at weaning/total number of pigs after cross-fostering] × 100. ^5^ Weaning weight was adjusted for weaning age by the following equation: body weight after cross-fostering + [(body weight at weaning—body weight after cross-fostering)/weaning age] × 21. ^6^ All OG × parity on test *p*-values were greater than 0.10, with the exception of a tendency for an interactive effect on stillborns (*p* = 0.099).

**Table 3:** Main effects of OmniGen-AF supplementation of sow diets on milk composition of sows from the University of Kentucky ^1^.

	Lactation Period ^2^		SEM ^4^	*p*-Value	OG ^3^		SEM ^4^	*p*-Value ^6^
Item	Early	Late			0%	0.75%		
No. of observation	145	138			152	131		
Fat, %	6.37	5.73	0.118	<0.001	6.17	5.92	0.121	0.133
Protein, %	4.92	4.54	0.032	<0.001	4.77	4.68	0.033	0.047
Lactose, %	5.70	5.92	0.021	<0.001	5.78	5.84	0.022	0.057
Total solids, %	18.01	17.13	0.130	<0.001	17.72	17.42	0.134	0.102
Non-fat solids, %	10.97	10.79	0.026	<0.001	10.89	10.86	0.027	0.409
Gross energy, MJ/kg ^5^	4.64	4.38	0.045	<0.001	4.56	4.46	0.046	0.126

^1^ Data are least squares means of milk composition of sows fed either 0% or 0.75% OG-supplemented diet. ^2^ Early and late lactation milk samples were collected during d 5 to 7 and d 14 to 17, respectively, from teats 3 and 4 on the sow’s right side. ^3^ OmniGen-AF (Phibro Animal Health, Fairfield, NJ) was included in diets at 0 or 0.75%. ^4^ SEM = standard error of the mean. ^5^ The gross energy content of the complete milk was calculated from the concentration of lactose, fat, and protein, which contributed 16.4, 38.9, and 23.8 KJ/g, respectively (Ramanau et al., 2004 [10]). ^6^ There were no effects (*p* > 0.10) of OG × stage of lactation on any response measure.

## Data Availability

Dataset available upon reasonable request from the authors.
